# Evaluation of probiotic properties and genome analysis of the new *Pediococcus acidilactici* strain 46A isolated from Chinese young adults: *in vitro* and *in vivo* studies

**DOI:** 10.3389/fmicb.2026.1725578

**Published:** 2026-02-16

**Authors:** Polina Makarycheva, Vincenzo Bellitto, Aristide Toussaint Nguele, Chiara Salvesi, Matteo Mozzicafreddo, Anna-Rita Attili, Dennis Fiorini, Daniele Tomassoni, Stefania Silvi, Cristina Miceli

**Affiliations:** 1 School of Biosciences and Veterinary Medicine, University of Camerino, Camerino, Italy; 2 Institut Supérieur des Sciences de la Santé, Université Adventiste Cosendai, Nanga Eboko, Cameroon; 3 Department of Clinical and Molecular Sciences, Marche Polytechnic University, Ancona, Italy,; 4 School of Sciences and Technologies, University of Camerino, Camerino, Italy

**Keywords:** bacterialgenome, chemical-induced colitis, gut microbiota, intestinal mucosa, lactic acid bacteria, probiotics

## Abstract

**Background:**

The human gut microbiota plays a key role in intestinal homeostasis and inflammatory diseases, prompting the search for novel probiotic strains with strain-specific functional properties. Differences in dietary habits and cultural backgrounds represent a valuable source for isolating previously uncharacterized gut bacteria. Given the increasing use of probiotics, rigorous safety assessment, including antimicrobial resistance (AMR) evaluation, is required, and the integration of whole-genome sequencing with phenotypic characterization is recommended.

**Objectives:**

This study evaluated the probiotic potential of *Pediococcus acidilactici* strain 46A, isolated from fecal samples of healthy Chinese student volunteers, through whole-genome analysis and *in vitro* and *in vivo* functional assessments.

**Methods:**

Strain identification was performed by MALDI-TOF mass spectrometry and 16S rRNA gene sequencing. *In vitro* assays assessed tolerance to simulated gastrointestinal conditions, bile salt resistance, antimicrobial activity, and adhesion to HT-29 intestinal epithelial cells. Whole-genome sequencing was used to evaluate safety- and functionality-related genetic traits. The protective effect of *P. acidilactici* 46A was investigated in a dextran sulphate sodium (DSS)-induced murine model of colitis, including histological evaluation, inflammatory marker analysis, and gut microbiota profiling.

**Results:**

*P. acidilactici* 46A showed high survival under simulated gastrointestinal conditions and bile exposure, antimicrobial activity against Gram-positive and Gram-negative bacteria and yeasts, and strong adhesion to HT-29 cells compared with other tested strains. Genomic analysis confirmed the absence of virulence factors and clinically relevant antibiotic resistance genes. In DSS-treated mice, oral administration of *P. acidilactici* 46A significantly reduced colitis severity, preserved colonic mucosal architecture, and decreased inflammatory markers, including IL-1β and IL-6.

**Conclusion:**

*Pediococcus acidilactici* 46A is a safe and functionally active probiotic candidate that attenuates intestinal inflammation and preserves mucosal integrity in a murine model of colitis, supporting its potential application in the prevention or adjunctive treatment of inflammatory gut disorders.

## Graphical abstract

**Figure:**
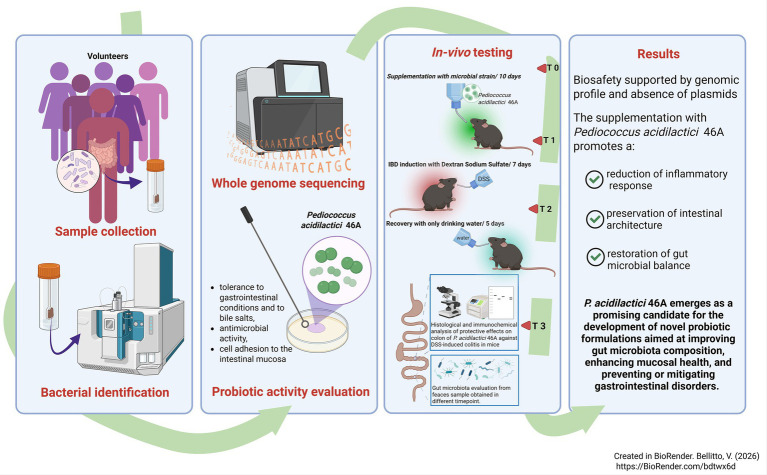

## 1 Introduction

The criteria for the selection of probiotics, including resistance to unfavorable conditions that the human body imposes, intestinal epithelium adhesion ability, anti-microbial activity, and safety assessment, are still valid (Butt et al., 2025; Irfan et al., 2025). The accumulation of evidence demonstrating the relationship between microorganisms and human health has generated additional strategies for selecting probiotics. These include methods to evaluate the biological properties of probiotics such as anticarcinogenic, antidepression, anti-obesity, anti-diabetic, anti-inflammatory and cholesterol-lowering activities, as well as safety aspects related to virulence, toxicity, and transferable antibiotic resistance (Sarita et al., 2025). Currently also neuroprotective effects, targeting key mechanisms of epileptogenesis have been evidenced (Sherwani et al., 2025). The evidence that probiotics modulate the cytokine production, strengthen the mucosal barrier, and reduce inflammation (Cristofori et al., 2021) suggests that they are effective in treating colitis (Côco et al., 2025). This aspect of probiotic activity is specifically important for understanding and treating inflammatory bowel diseases (IBD), including Crohn’s disease and ulcerative colitis (Estevinho et al., 2024).

The focus of this study was on the anti-inflammatory properties of the potential probiotic strain *Pediococcus acidilactici* 46A. This species is associated with dairy products, found in some cheeses, and occasionally used in the fermentation of milk and during cheese manufacture (Porto et al., 2017). Multiple studies indicated the probiotic capacity of *Pediococcus* species due to their ability to survive and adhere to the gastrointestinal tract and to report immune modulation capability (Fijan, 2014; Araújo et al., 2016; Jang et al., 2021; Tabashiri et al., 2025). It was also observed that *P. acidilactici* could be an effective probiotic for hypocholesterolemia effects (Sudun et al., 2019), blood glucose regulation, thereby combating diabetes and obesity (Yavorov-Dayliev et al., 2023). Some strains have antimicrobial activity, by producing bacteriocins (pediocins) (Jiang et al., 2021; Liu et al., 2024). Tong et al. (2020) recorded that *P. acidilactici* was able to inhibit the *in vitro* growth of food-born *Escherichia coli* K88, *Staphylococcus aureus*, and *Salmonella* spp. (Tong et al., 2020). Despite the availability of multiple *in vitro* and *in vivo* studies on *P. acidilactici* strains, little information is available on its *in vivo* safety and performance in conditions of induced colitis. This study aimed to characterize and study the new bacterial strain *P. acidilactici* 46A, its probiotic potential, and anti- inflammatory characteristics by application of whole-genome sequencing, standard tests to assess the probiotic properties of the strains, and *in vivo* tests on mice with dextran sodium sulphate induced colitis. Dextran sulfate sodium (DSS)-treated mice were chosen as a well-characterized animal model of colitis that allows for a comprehensive analysis of colitis status and the possible preventive effects of different treatments (Thallmair and Jirkof, 2025). Chemically induced models of colonic inflammation are the most common pre-clinical approach to study inflammatory bowel disease. The administration of DSS in drinking water establishes acute colitis that can be evaluated in *ex vivo* samples through histological approaches (Kim et al., 2012). The results of this study will provide a better understanding of the anti-inflammatory activity of selected bacterial strain.

## 2 Materials and methods

### 2.1 Strain selection, culturing, and species identification

During practical activities involving healthy student volunteers from Jilin Agricultural University (Changchun, China), who were studying at the University of Camerino as part of a double degree program, several bacterial strains were isolated from stool samples provided by the students themselves in the first few days after their arrival in Italy. The students were clearly informed about the purpose of the practical activity, which was to teach them how to isolate new probiotics, and about the use of the samples. The samples were used in the practical exercise without identifying each provider, and they were destroyed after bacteria selection.

The approval of the Ethics Committee of the University of Camerino about the use of biological material for this study was obtained (UNICAM E. C. - Prot. n. 0095481, 19/11/2025).

Aliquots of fecal samples, suspended in saline solution, were streaked onto de Man, Rogosa, and Sharpe Agar (MRS- Liofilchem®, Italy) (Verdenelli et al., 2009; Coman et al., 2019) and cultured at 36 ± 1 °C for 24–48 h in the anaerobic atmosphere (AnaeroGen Gas Generating System, ThermoFisher, Italy). From the selective agar plates the colonies that appeared with different morphology were picked up and observed microscopically. The strains that stained Gram positive and showed cell morphology compatible with lactic acid bacteria were selected. Nine strains were chosen and identified (Supplementary Table S1). MALDI-TOF MS (Bruker Microflex Lt®, Bruker Daltonics, Germany) was used for the strain identification. Following the manufacturer’s instructions, the Standard Operating Procedure - Direct Transfer Procedure (SOP Direct Transfer Procedure Revision.4) - was used. Briefly, the bacterial colony was first inoculated in a MALDI-TOF MS target plate, and subsequently, 1 μL of *α*-Cyano-4-hydroxycinnamic acid matrix solution (Bruker Matrix HCCA) was added to the sample and dried at room temperature for 10 min. For the analysis, mass spectra were processed using Flex Analysis (version 3.4; Bruker Daltonics, Germany) and BioTyper software (version 3.1; Bruker Daltonics, Germany). The identification was based on the score values released by the equipment’s instructions. Specifically, log (score) below 1.700 indicated a non-reliable identification, between 1.700 and 1.999 a probable genus identification, while a log (score) of ≥2.000 indicated a reliable genus identification and a highly probable species-level identification. The row spectra obtained were compared with those present in the Biotyper database and log (score) ≥ 2.000 was considered. A bacterial test standard (BTS) (Bruker Daltonics, Germany) was used as a calibrator for quality control. *P. acidilactici* strain 46A, identified by MALDI-TOF MS, was further confirmed by 16S rDNA analysis, and finally selected among the others to be tested on mice, since it showed good probiotic properties.

### 2.2 Preliminary test for probiotic properties

The probiotic candidate strains were tested for tolerance to gastrointestinal conditions and to bile salts, antimicrobial activity, and cell adhesion to the intestinal mucosa.

The acid tolerance assay was performed by inoculating each bacterial suspension (10^8^ CFU/mL) in acidic MRS broth (pH 3.0) and incubating at 37 °C in aerobic conditions. Viable bacterial cell counts (CFU/ml) were performed after 0, 1, 2, 3, 4 and 5 h of incubation to assess the survival percentage of the 9 probiotic candidates (Supplementary Figure S2).

The same bacterial suspension (10^8^ CFU/mL) was also used for bile acid tolerance assay. Viable bacterial cell counts were conducted onto MRS agar plates, enriched with 0.1, 0.3, 0.5% of Bile salts (Oxoid, Hampshire, UK) and onto MRS agar plates with 0.05, 0.1, 0.2% of Bile salt N.3 (Oxoid). MRS agar plates used as control. CFU/ml were enumerated after 24/48 h incubation of plates at 37 °C giving the percentage of survival in presence of purified and conjugated bile acids (Supplementary Figure S3).

The antimicrobial activity assay was performed by Radial streak test for the five bacterial strains (Supplementary Figure S4) that had the best combined results from the previous tests.

*P. acidilactici* 46A exhibited antimicrobial activity against Gram-positive and -negative bacteria and yeasts when tested against the following: *Bacillus cereus* DSM 345*, Escherichia coli* ATCC 13706*, Salmonella enterica* DSM 14221*, Staphylococcus aureus* ATCC 25923*, Pseudomonas aeruginosa* DSM 1117*, Listeria monocytogenes 306, Enterococcus faecium* DSM 13590*, Candida albicans* ATCC 10261 (Supplementary Figure S4).

Four out of last five bacterial strains were tested for the adherence assay to intestinal epithelial HT-29 cell lines (Verdenelli et al., 2014; Supplementary Figure S5). *P. acidilactici* 46 A exhibited the highest percentage of adhesion to intestinal epithelial cells compared to the other three strains. In conclusion, in light of all the tests, *P. acidilactici* 46 A proved to be the best probiotic candidate.

### 2.3 Antibiotic susceptibility testing

Subsequently *P. acidilactici* 46A was tested against a panel of 41 human and veterinary antibiotics (Liofilchem®, Teramo, Italy), belonging to 21 categories: penicillins (benzylpenicillin G P-1UI, ampicillin AMP-10 μg, piperacillin PRL-30 μg, oxacillin OX-1 μg); penicillins+beta-lactamase inhibitors (amoxicillin and clavulanate acid AUG-30 μg); carbapenems (imipenem IMI-30 μg); I gen. Cephalosporins (cefadroxil CDX-30 μg, cephalexin CL-30 μg); II gen. Cephalosporins (cefoxitina FOX-30 μg, cefixime CFM-30 μg, ceftiofur FUR-30 μg,); III gen. Cephalosporins (ceftazidime CAZ-30 μg); IV gen. Cephalosporins (cefquinome CEQ-30 μg); monobactams (aztreonam ATM-30 μg); fluoroquinolones (ciprofloxacin CIP-5 μg, enrofloxacin ENR-30 μg, norfloxacin NOR-10 μg, nalidix acid NA-30 μg); aminoglycosides (amikacin AK-30 μg, gentamicin CN-10 μg, tobramycin TOB-10 μg); glycopeptides (vancomycin/teicoplanin MIC E-test VA/TEC 0.5–32/0.5-32 μg/mL); macrolides (azithromycin AZM-15 μg, erythromycin E-15 μg, streptomycin S-300 μg, spiramycin SP-100 μg); lincosamides (clindamycin CD-2 μg); streptogramins (quinupristin-dalfopristin QD-15 μg); tetracyclines (tetracycline TE-30 μg; tigecycline TGC-15 μg); polymyxins (colistin MIC E-test CS 0.016-256 μg/mL; polymyxin B PB-300 IU); oxazolidones (linezolid LNZ-10 μg); folate pathway inhibitors (sulfametoxazole and trimethoprim SXT-25 μg); amphenicols (florfenicol FFC-30 μg); phosphonic antibiotics (fosfomycin FOS-200 μg); rifamycins (rifampicin RD-5 μg); lipopeptydes (daptomycin DAP-30 μg); mupirocins (mupirocin MUP-200 μg); and nitrofurans (nitrofurantoin F-100 μg). Kirby-Bauer and MIC E-test methods were performed on MRS agar plates (Liofilchem®, Teramo, Italy), following European Committee on Antimicrobial Susceptibility Testing recommendations (European Committee on Antimicrobial Susceptibility Testing, 2024). Isolates that exhibited intermediate susceptibility were considered susceptible to the antibiotic as suggested by EUCAST guidelines (European Committee on Antimicrobial Susceptibility Testing, 2021).

### 2.4 Whole-genome sequencing of *P. acidilactici* 46A

For sequencing purposes, DNA was extracted from overnight cultures of *P. acidilactici* 46A cultured in LB medium. For extraction, the Power Soil kit from Qiagen was used and DNA was quantified by Qubit 4.0 Fluorimeter (Thermo Scientific, Waltham, MA, USA). The DNA library was prepared according to the Illumina DNA Prep Guide for Illumina Paired-End Indexed Sequencing. DNA sequencing was performed by Synbiotec laboratory (total output: 261 Mb, estimated genome size: 2.08 Mb and estimate genome coverage of 125X) (Camerino, MC, Italy). The genome was assembled using Unicycler (Version 0.4.8). Assessment of genome assembly quality was performed using Quast (Version 5.2.0). The complete genome was annotated using the Prokaryotic Genome Annotation System (Prokka - v1.14.6).

Genes were analyzed using Cluster Orthologous Groups (COG) (Galperin et al., 2021) with the Kyoto Encyclopedia of Genes and Genomes (KEGG) (Kanehisa et al., 2022). Antibiotic resistome analysis was performed using the Comprehensive Antibiotic Resistance Database (CARD) (Alcock et al., 2020). Proksee was used for whole genome circular visualization (Grant et al., 2023). MinCED (version 0.2.0)^1^ was used to identify CRISPR sequences. COG classifier^2^ was used to prepare data representing COG of protein functional classification. Phylogenetic tree was constructed using MEGA11 software (Tamura et al., 2021).

### 2.5 Experimental design of the *in vivo* study

**Figure 1:**
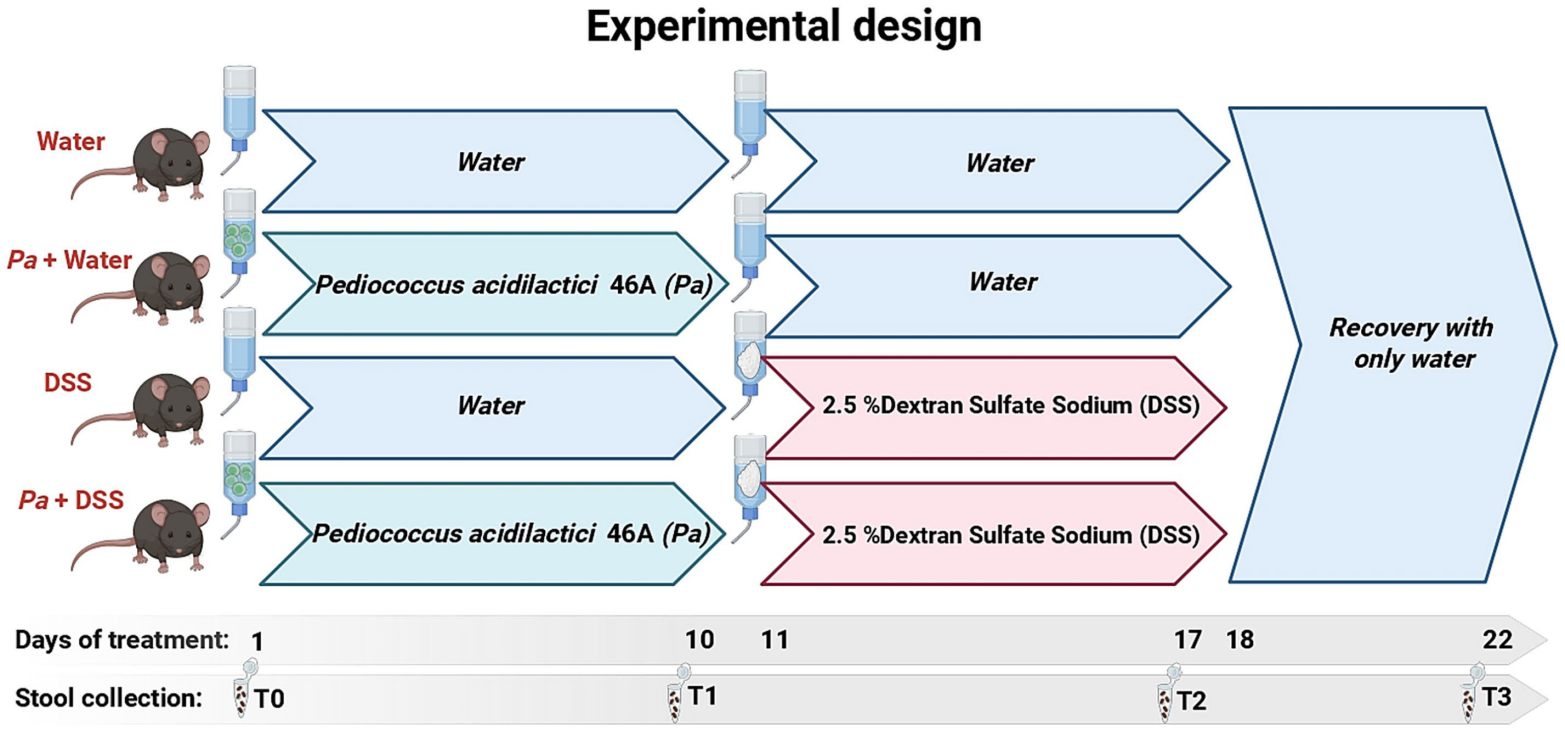
Timeline of the experiment, including duration of the experiment in days and corresponding time points. Created in BioRender. Bellitto, V. (2026), https://BioRender.com/gr92090.

In this preliminary study (Authorization of Italian Ministry of Health n° 362/2022-PR, Risp. a prot. 1D580.37 24/06/2022), for evaluating the probiotic effect of *P. acidilactici* 46A, 8-week-old male mice (C57BL/6 J) were used as animal model of DSS-induced colitis. Thirty-two mice were purchased from Inotiv (Italy) and were housed in a 12-h light/12-h dark cycle (lights on at 9:00 a.m.) with access to food (4RF25 Mucedola, Settimino Milanese, MI, Italy) and water *ad libitum* for 2 weeks before the experiments, in a room at constant temperature (20–22 °C) and humidity (45–55%), following the ARRIVE reporting guidelines (Percie du Sert et al., 2020). Mice, with no significant differences in body weight, were randomly divided into four groups in accordance with the experimental design (Figure 1).

- Control/water group: mice were provided with drinking water for 22 days (n = 8).
- *Pa*+ water group: mice were provided with probiotic *P. acidilactici* 46A (1×10^8^ CFU/die) dissolved in drinking water from day 1 to day 10, hence with drinking water from day 11 to day 22 (n = 8).
- DSS group: mice were provided with drinking water from day 1 to day 10, hence with 2.5% w/v DSS (Colitis grade MW 36,000-50,000; MP Biomedicals, Cat. No. 160110) solution from day 11 to day 17 and again drinking only water from day 18 to day 22 (n = 8).
- *Pa*+ DSS group: mice were provided with probiotic *P. acidilactici* 46A (1×10^8^ CFU/die) dissolved in drinking water from day 1 to day 10, hence with 2.5% DSS solution from day 11 to day 17, and again only drinking water from day 18 to 22 (n = 8).

G* power analysis (*α*-error of 0.05, power 0.9) considering the colitis score and clinical symptoms was used to evaluate the sample size of each group. As reported above, the experimental framework provides a supplementation of the probiotic for 10 days, from day 1 to day 10, to groups *Pa*+ water and *Pa*+ DSS. For this treatment *P. acidilactici* 46A was administered at a dose of 10^8^ probiotic cells/day and prepared daily to prevent differences in vitality. Oral administration was chosen to reproduce the way of administration in humans and to reduce at the minimum animal damage, being a non-invasive method. Stools sampling was performed by placing a single mouse in an empty cage without bedding for 15–30 min. With sterile forceps, the feces were collected in a microfuge tube and frozen at −20 °C. In review.

Fecal samples were collected at defined time points (Figure 1):

- T0, before any intervention;
- T1, after 10 days of probiotic supplementation in the Pa + water and Pa + DSS groups;
- T2, after 7 days of DSS treatment in the DSS and Pa + DSS groups, while the Pa + water group was undergoing a post-probiotic adaptation period with drinking water;
- T3, after a 5-day recovery period with pure water following DSS treatment.

In order to monitor the severity of colitis, body weight trend was daily monitored and stools consistence was evaluated every day and classified according to the indications of a scale with a score of 0–3 (Chassaing et al., 2014); normal stool consistency with negative occult blood: 0; loose stools with positive blood count: 1; very loose stools with traces of blood: 2; watery stools with visible rectal bleeding: 3. All the animals treated with a DSS showed clinical symptoms of colitis. All fecal samples were stored at −20 °C. Stool samples were collected on the first day before starting the treatment and then every 7 days. Figure 1 represents a more visual description of the timeline of the experiments.

### 2.6 Histological analysis on the mice gut

At the end of the experimental period, the animals were euthanized in a chamber by CO_2_ gradual-fill. All the intestinal tract was removed. The colon was measured using a graduated ruler from the ileocecal junction at the terminal end of the rectum. The fecal contents were removed with forceps and by flushing the intestine with cold phosphate buffer saline (PBS) solution, 10 mM pH 7.4, using a blunt needle attached to a syringe. The proximal and distal portions of each colon were divided into two parts: one was stored immediately at −80 °C for biochemical analysis, while the second was fixed in 4% paraformaldehyde solution buffered with PBS and successively, according to the protocol, the samples were dehydrated in grading alcohol, clarified in xylene, and embedded in paraffin wax.

#### 2.6.1 Morphological analysis

Consecutive cross sections with a thickness of 6 μm were deparaffinized in xylene two times for 10 min each and rehydrated at room temperature with progressively lower grades of ethanol (starting from 100% down to 70%) and washed in distilled water. After complete rehydration, the slides were stained alternatively with Masson’s Trichrome to evaluate the morphology and pattern of fibrosis, with Periodic Acid-Schiff staining (PAS) and with Alcian Blue pH 2.5, to assess the mucus pattern of the glands.

After mounting, sections were analyzed with the computer-assisted semi-quantitative method: images were reported from a light microscope (Leica DMR, Germany) by using a camera (Nikon DS-Ri2, Japan) to an image analysis software (Nikon Nis Element, Japan). The histological damage was calculated by a system of scores which considers alterations of the crypt architecture, the presence of inflammatory infiltrate, the thickening of the tunica muscularis and the depletion in goblet cells, using the following system: crypt architecture (normal, 0 - severe crypt distortion with loss of entire crypts, 3), degree of inflammatory cell infiltration (normal, 0 - dense inflammatory infiltrate, 3), muscle thickening (base of crypt sits on the muscularis mucosae, 0 - marked muscle thickening present, 3), goblet cell depletion (absent, 0- present, 1) and crypt abscess (absent, 0- present, 1). The histological damage score was calculated as the sum of each individual score.

#### 2.6.2 Immunohistochemistry

Alternative colon sections were exposed overnight at 4 °C to Interleukin-1 beta (IL-1β) (Cod. AAR15G, Bio-Rad Laboratories, Inc., California, USA) and Interleukin-6 (IL-6) (Cod. GTX-110527 GeneTex, Hsinchu, Taiwan, ROC) antibodies diluted in 1X PBS/0.3% Triton X-100 (PBS/Triton) (200 μL per section). The product of the immune reaction was then revealed by incubating slides for 30 min at 25 °C with the specific biotinylated secondary IgGs (Bethyl Laboratory, Inc., Montgomery, TX, USA) at a dilution of 1:200 in PBS/Triton. The immune reaction was then highlighted with 3, 3′-diaminobenzidine (Cod. SK-4100 Vector Laboratories, Inc., Burlingame, CA, USA) as a substrate. Sections were also counterstained with hematoxylin and then washed, dehydrated in progressively higher concentration of ethanol, clarified in xylene, mounted on cover slips, and viewed under a light microscope. To assess the immunostaining background, a group of sections were incubated with a non-immune serum instead of a primary antibody. After mounting, sections were recorded as previously detailed.

#### 2.6.3 Western blot

The protein expression of pro-inflammatory cytokines like IL-1β (Cod. AAR15G, Bio-Rad Laboratories, Inc., California, USA) and IL-6 (Cod. sc-57315 Santa Cruz Biotechnology, Inc. Dallas, USA) antibodies evaluated using the Western blot technique. Samples of proximal and distal colon stored in −80 °C were homogenized in lysis buffer composed of Tris 1 M pH 7.4, NaCl 1 M, EGTA 10 mM, NaF 100 mM, Na_3_VO_4_ 100 mM, PMSF 100 mM, Deoxycholate 2%, EDTA 100 mM, Triton X100 10%, Glycerol, SDS 10%, Na_4_P_2_O_7_ 0.1 M, Protease Inhibitor Cocktail, distilled H_2_O and the collected supernatants were evaluated to determine the protein concentration by Bradford assay, in accordance with protocols previously used (Bellitto et al., 2024). Forty μg of proteins were separated in 12% Sodium Dodecyl Sulphate-Polyacrylamide Gel Electrophoresis (SDS-PAGE) and transferred into a nitrocellulose membrane. Membranes were probed overnight at 4 °C with the primary antibodies: IL-1*β* and IL-6 diluted in 1X PBS containing Tween 20 (1:5000). Then, the immunoreaction was amplified at room temperature with a specific horseradish peroxidase (HRP)-conjugated secondary antibodies (Bethyl Laboratories, Inc., Montgomery, Texas, USA) diluted in 1X PBS containing Tween 20 (1:5000). The immunoreaction products were revealed using the Lite blot Plus and Turbo kits (Euroclone, Milan, Italy). The optical density and relative’s bands intensities were determined through Bio-Rad image analysis (Bio-Rad Laboratories, Inc., California, USA). For quantification, β-actin (Cod. A2228, Merck-Millipore Co., Darmstadt, Germany) was used as a protein loading control.

#### 2.6.4 Gut microbiota analysis on mice stool samples

All fecal samples, stored at −20 °C, were used for the extraction of bacterial DNA.

The fecal samples collected at each timepoint as specified in Figure 1 were extracted using QIAamp Fast DNA Stool Mini kit (Qiagen) following the instructions provided by the producer. The quality and quantity of extracted DNA were controlled by The NanoDrop™ microvolume sample retention system (Thermo Scientific NanoDrop™ Products).

Polymerase chain reaction for validation and quality control of DNA was performed following the protocol described previously (Krych et al., 2018), with slight modifications. Specific primers for the V3-V4 hypervariable region of the 16 s rRNA were used:

Forward Pro341F: 5′TCGTCGGCAGCGTCAGATGTGTATAAGAGACAGCCTACGGGNBGCASCAG-3′, reverse Pro805R: 5′GTCTCGTGGGCTCGGAGATGTGTATAAGAGACAGGACTACNVGGGTATCTAATCC-3′.

PCR was performed at 50 UL volumes containing 2.5 μL of TaqBuffer (Pyrobest DNA Polymerase (TaKaRa)), 1 μL forward primers, 1 μL reverse primers, 0.5 μL of dNTPs, 0.5 ul of TaqPolymerase enzyme, and 2 μL of DNA per sample. The PCR was programmed as follows: denaturation steps at 94 °C for 5 min, followed by 35 cycles of denaturation at 94 °C for 30 s, annealing at 53 °C for 30 s, extension at 72 °C for 90 s, and final extension step at 72 °C for 10 min.

Further on, all samples were run in an electrophoresis chamber on 1% agarose gel.

V3-V4 regions of 16S rRNA were sequenced using an Illumina Miseq (by Synbiotec Srl, Camerino, Italy). The sequencing depth was evaluated by generating a rarefaction plot for each sample. This plot is included in the supplemental data section as Supplementary Figure S1. The plateau in observed species is reached for each sample based on its sequencing depth.

Sequencing data were analyzed by the software QIIME2 (Quantitative Insights into Microbial Ecology, version 2023.5). Briefly, after filtering out low-quality reads (minimum quality score of 25, minimum/maximum length of 200–250, no ambiguous bases allowed, no mismatches allowed in the primer sequence, and no phiX reads/chimeric sequences), all remaining sequences were clustered into Operational Taxonomic Units (OTUs) on the basis of similarity following the DADA2 pipeline and using the QIIME 2 Plugin ‘dada2’ version 2023.5.0. Samples were evaluated for alpha diversity (microbial diversity within samples) and beta diversity (community divergence between samples) calculations in QIIME2.

### 2.7 Short chain fatty acid content in mouse fecal samples

An aliquot of the fecal samples from the control group mice and the mice treated with probiotics, corresponding to the first and second lines of Figure 1, indicated as “Water” and “*Pa* + Water”, was used for short-chain fatty acids (SCFA) analysis. The method consisted of extraction of the SCFAs (acetic, propionic, i-butyric, butyric, i-valeric, valeric, i-caproic and caproic acids) by ethyl ether after acidification of the samples. The SCFAs extracted were directly analyzed by GC-FID without derivatization and separated on a polyethylene glycol nitroterephthalic acid modified coated capillary column, following the procedures described previously (Salvesi et al., 2022).

### 2.8 Statistical analysis

*Ex vivo* data are expressed as mean ± standard error of the mean (S. E. M) and were analyzed by one-way analysis of variance (ANOVA) and a non-parametric Kruskal–Wallis test using GraphPad Prism Software (Version 6.01), followed by *post hoc* comparison (Tukey test). The *p*-values < 0.05 were considered to be statistically significant. Alpha diversity metrics were assessed for statistical significance using a two-sample *t*-test and a Kruskal–Wallis test as implemented in QIIME2. Beta diversity metrics were assessed using the adonis2/PERMANOVA statistical test.

Taxonomic analysis was performed by matching OTU sequences with both the Silva and Greengenes databases.

## 3 Results

### 3.1 *Pediococcus acidilactici* 46A whole genome sequencing

**Figure 2:**
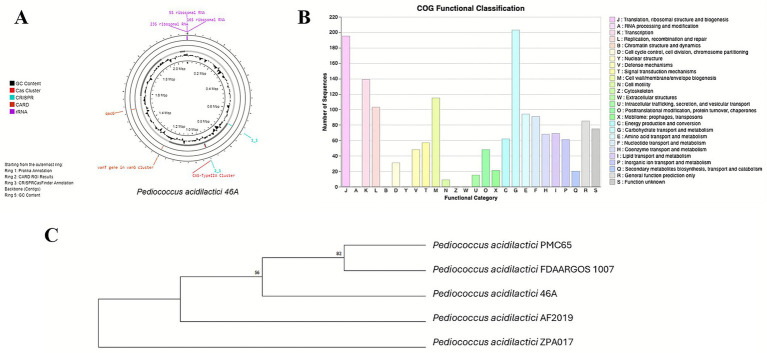
**(A)** Circular genome of *Pediococcus acidilactici* 46A. **(B)** COG of protein functional classification *P. acidilactici* 46A. **(C)** Neighbor-joining phylogenetic tree *P. acidilactici* 46A.

*Pediococcus acidilactici* 46A was selected as a probiotic candidate because it showed the best characteristics of pH and bile acid resistance, and antimicrobial activity against Gram positive and negative bacteria, and yeasts. In addition, this strain exhibited the highest percentage of adhesion to intestinal epithelial cell models with respect to the other studied strains (Supplementary Figure S5). The whole genome sequencing of *P. acidilactici* 46A revealed a total genome length of 2,086,432 bp, with GC% content equal to 42.09%. The *P. acidilactici* 46A genome contains a total of 2073 genes with an average length of 885 bp. The total length of coding sequences is 1,833,094 bp, corresponding to 87.85% of the total genome length (Figure 2A). Five CRISPR regions were identified and reported in Table 1.

**Table 1:** Minced software analysis revealed the presence of 5 CRISPR sequences.

CRISPR regions in *P. acidilactici* 46A genome	Range	Repeats
CRISPR1	76–218	3
CRISPR2	1,195–1893	9
CRISPR3	1,121–1,526	6
CRISPR4	43–580	7
CRISPR5	1956–2,143	3

KEGG database analysis revealed 883 coding sequences associated with various metabolic pathways. CARD: RGI database analysis revealed three strict hits referring to genes ARO term qacG, sdrM, vanT gene in vanG cluster (Table 2). COG of proteins functional classification showed the distribution of proteins and their functions as in Figure 2B.

**Table 2:** CARD database search based on the whole genome sequence of *Pediococcus acidilactici* 46A, reporting only the strict hits in the parameter of search, heading expressing the ARO term, and detection criteria based on protein homolog model.

	sdrM	VanT gene in VanG cluster	qacG
AMR gene family	Major facilitator superfamily (MFS) antibiotic efflux pump	Glycopeptide resistance gene cluster	Small multidrug resistance (SMR)
Drug class	Fluoroquinolone antibiotics, disinfecting agents, and antiseptics	Glycopeptide antibiotic	Disinfecting agents and antiseptics
Resistance mechanism	Antibiotic efflux	Antibiotic target alteration	Antibiotic efflux
% Identity of matching region	34.81	31.42	48.11
% Length of reference sequence	109.04	52.67	99.07

The 16S rDNA sequence of *P. acidilactici* 46A strain, taken from the genome sequence data, was compared to 16S rDNA sequences of the other *P. acidilactici* strains available in databases, including the sequence of the strain PMC65, reported as a reference genome in the NCBI Genome database. The derived phylogenetic tree is shown in Figure 2C; National Center for Biotechnology Information Taxonomy Browser (2025).

### 3.2 *Pediococcus acidilactici* 46A antimicrobial susceptibility testing

*Pediococcus acidilactici* 46A, was first identified by MALDI-TOF MS with a log (score) of 2.103. Colonies appeared smooth, opaque, and round opalescent. By optical microscope, cultures showed cocci (0.4–1.4 μm in diameter) in pairs, which divided in two planes to form tetrads, without flagella, and with a Gram-positive staining. Cocci.

The phenotypic antimicrobial susceptibility results are described in Supplementary Table S1. The strain tested resistant to: oxacillin (penicillins); imipenem (carbapenems); cefadroxil, cefoxitina, cefixime and ceftazidime considering the cephalosporins (I, II, III and IV generation); aztreonam (monobactams); all fluoroquinolones, aminoglycosides and glycopeptides tested; azithromycin and streptomycin (macrolides). It is even resistant to others, such as quinupristin-dalfopristin, tetracycline, colistin, polymyxin B, sulfametoxazole and trimethoprim, fosfomycin, rifampicin, daptomycin and mupirocin.

### 3.3 Histological and immunochemical analysis of protective effects of *Pediococcus acidilactici* 46A against DSS-induced colitis in mice

**Figure 3:**
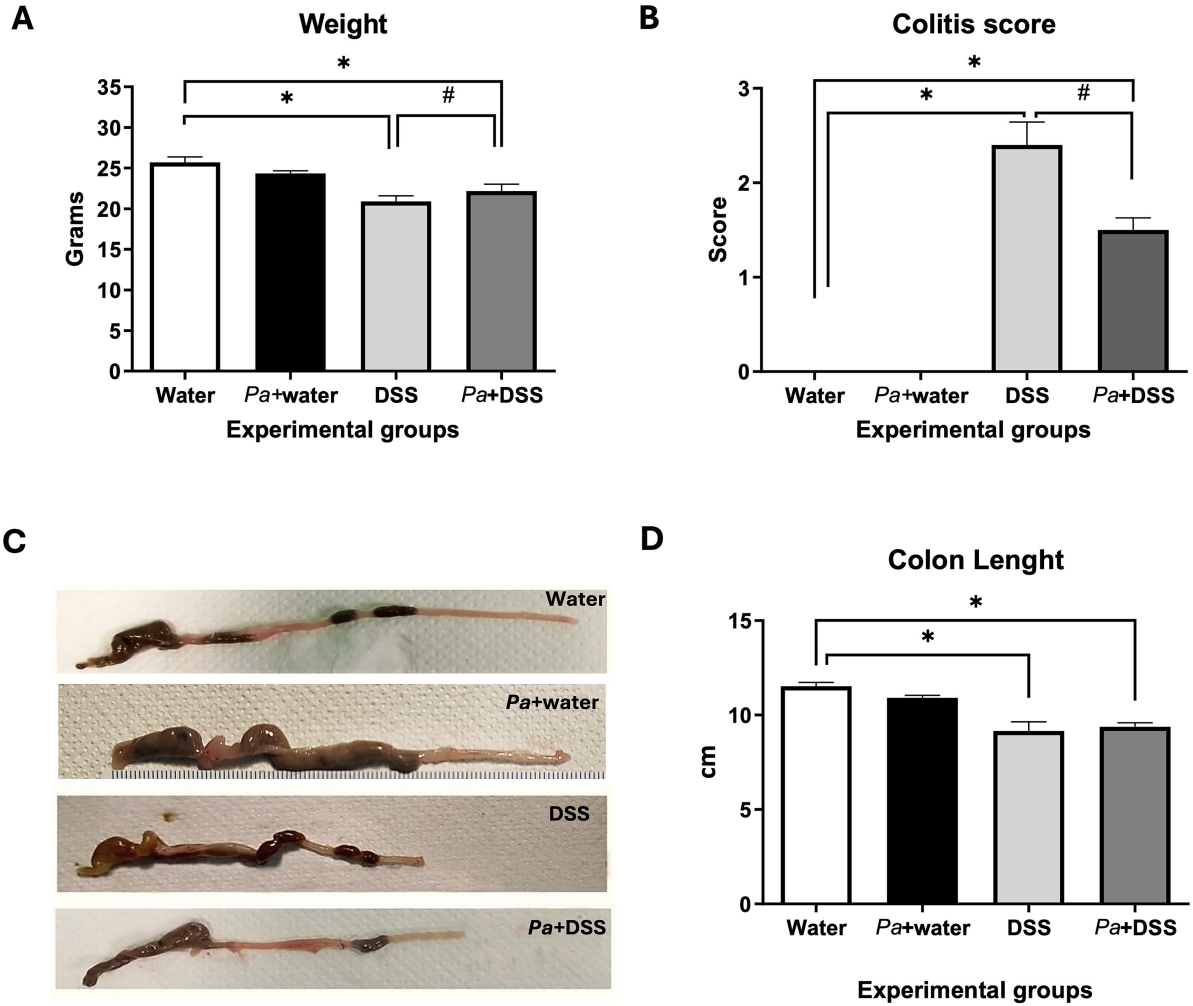
Mice weight in the different experimental groups **(A)**. Graph reporting the colitis score in the different experimental groups **(B)**. Representative photographs of colonic tract of a mouse for each experimental group **(C)** and relative graphs with colon length measurements **(D)**. Water: control mice; *Pa*+water: *P. acidilactici* 46A – pretreated control mice; DSS 2.5% dextran-sulphate sodium treated mice; *Pa*+ DSS: *P. acidilactici* 46 A – pretreated DSS. The score values are the mean ± SEM. *p < 0.05 *vs* water control mice: ^#^*p* > 0.05 *vs* DSS.

Clinical symptoms were assessed daily by checking the body weight changes of each mouse, evaluating the colitis score by monitoring the stools consistency and the presence of occult blood, checking the physical appearance and the behavioral response to external stimuli. Mice weight analysis showed a reduction of body weight in the DSS group compared to the control group; in the case of *P. acidilactici* 46A-pretreated DSS mice, the body weight was reduced compared to the control group and was similar to the DSS group, suggesting that the administration of the bacterial strain was not able to contrast the weight loss induced by DSS (Figure 3A). The results collected for each group were reported in Figure 3B. The observed results of the colitis score showed that the degree of colitis severity reported in the *Pa* + DSS group mice was significantly reduced with respect to the data observed for the DSS group (Figure 3B). After the sacrifice, the measurement of the colonic tract showed a reduction of colon length (Figure 3C) in the DSS group compared to the control group; in the *P. acidilactici* 46A-pretreated DSS mice, the length was reduced compared to the control group and resulted similar to the DSS group, suggesting that the administration of *P. acidilactici* 46A was not able to contrast the colonic shortening induced by DSS (Figure 3D).

**Figure 4:**
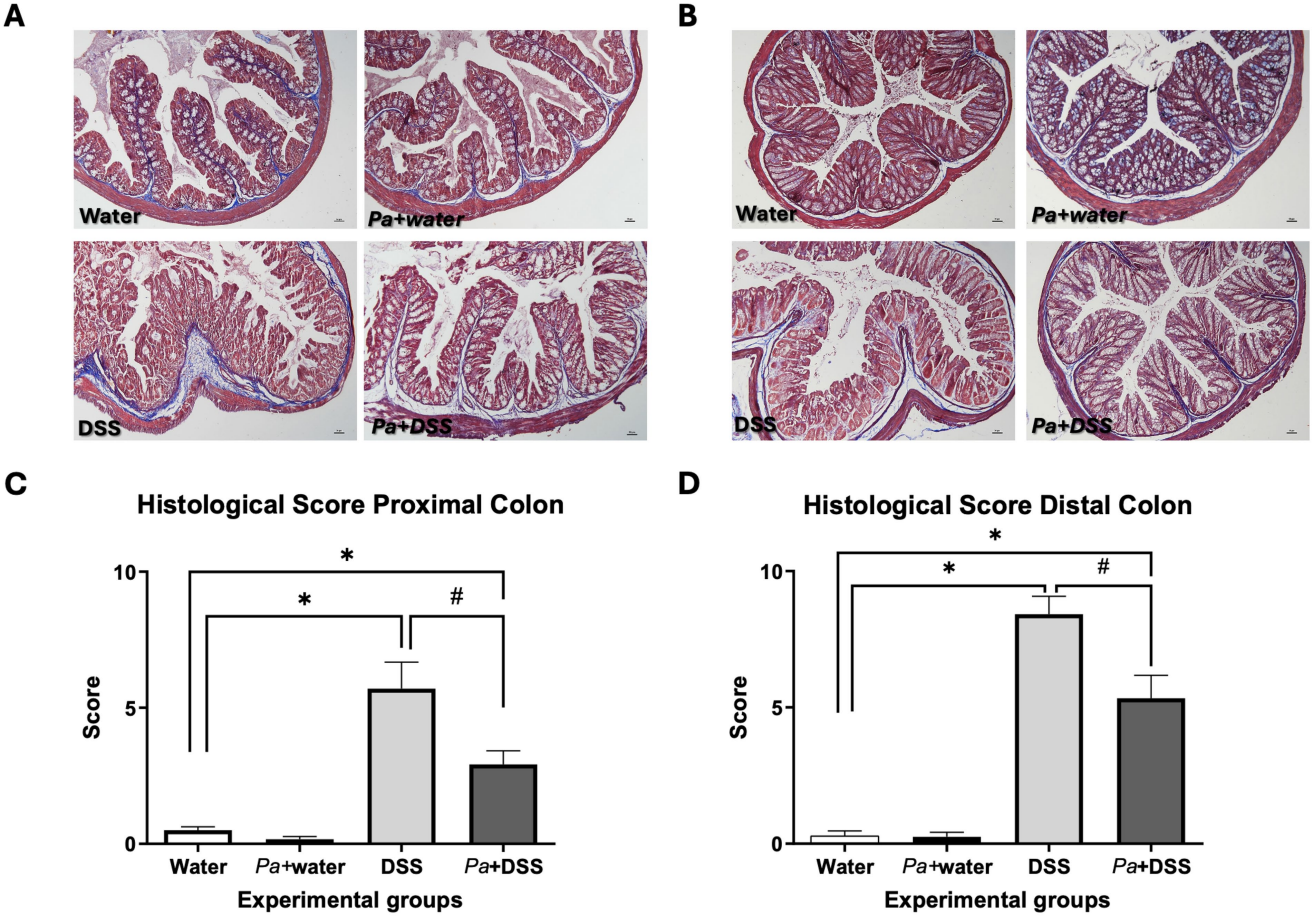
Representative sections of proximal **(A)** and distal colon **(B)** of mice belonging to different experimental groups, stained with Masson’s Trichrome technique. Calibration bar: 100 μm. The graphs report the histological score index for the proximal **(C)** and distal colon **(D)**. Water: control mice; *Pa*+water: *P. acidilactici* 46A – pretreated control mice; DSS: 2.5% dextran-sulphate sodium treated mice; *Pa*+ DSS: *P. acidilactici* 46A-pretreated DSS mice. The score values are the mean ±SEM. *p < 0.05 *vs* water control mice; ^#^*p* < 0.05 *vs* DSS.

**Figure 5:**
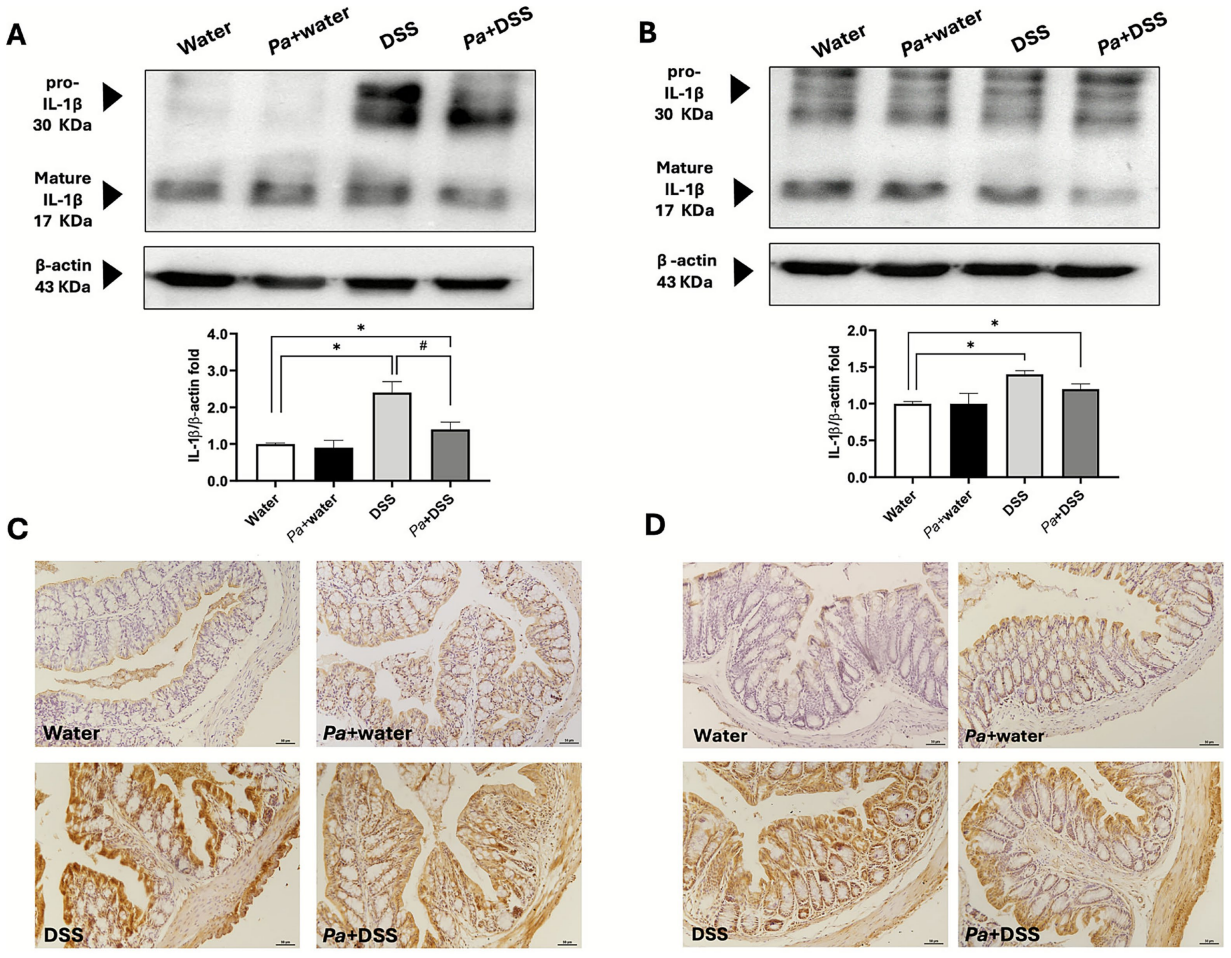
Interleukin 1 beta (IL-1*β*) expression level evaluation in western blot and immunohistochemical technique. A specific antibody against IL-1β was immunoblotted on a lysate of proximal **(A)** and distal colon **(B)**. The relatives’ graphs indicate the ratio of the densitometric analysis of pro-and mature form of IL-1β bands and β-actin levels were used as reference loading control [bottom line of panels **(A,B)**]. Representative sections marked with anti- IL-1β of proximal **(C)** and distal colon **(D)** of mice belonging to the different experimental groups, as indicated at the bottom left of each figure. Calibration bar: 50 μm. Water: control mice; *Pa*+water: *P. acidilactici* 46A-pretreated control mice; DSS: 2.5% dextran-sulphate sodium treated mice; *Pa*+DSS: *P. acidilactici* 46A-pretreated DSS mice. The score values are the mean ± SEM. *p < 0.05 *vs* water control mice; ^#^*p* < 0.05 *vs* DSS.

**Figure 6:**
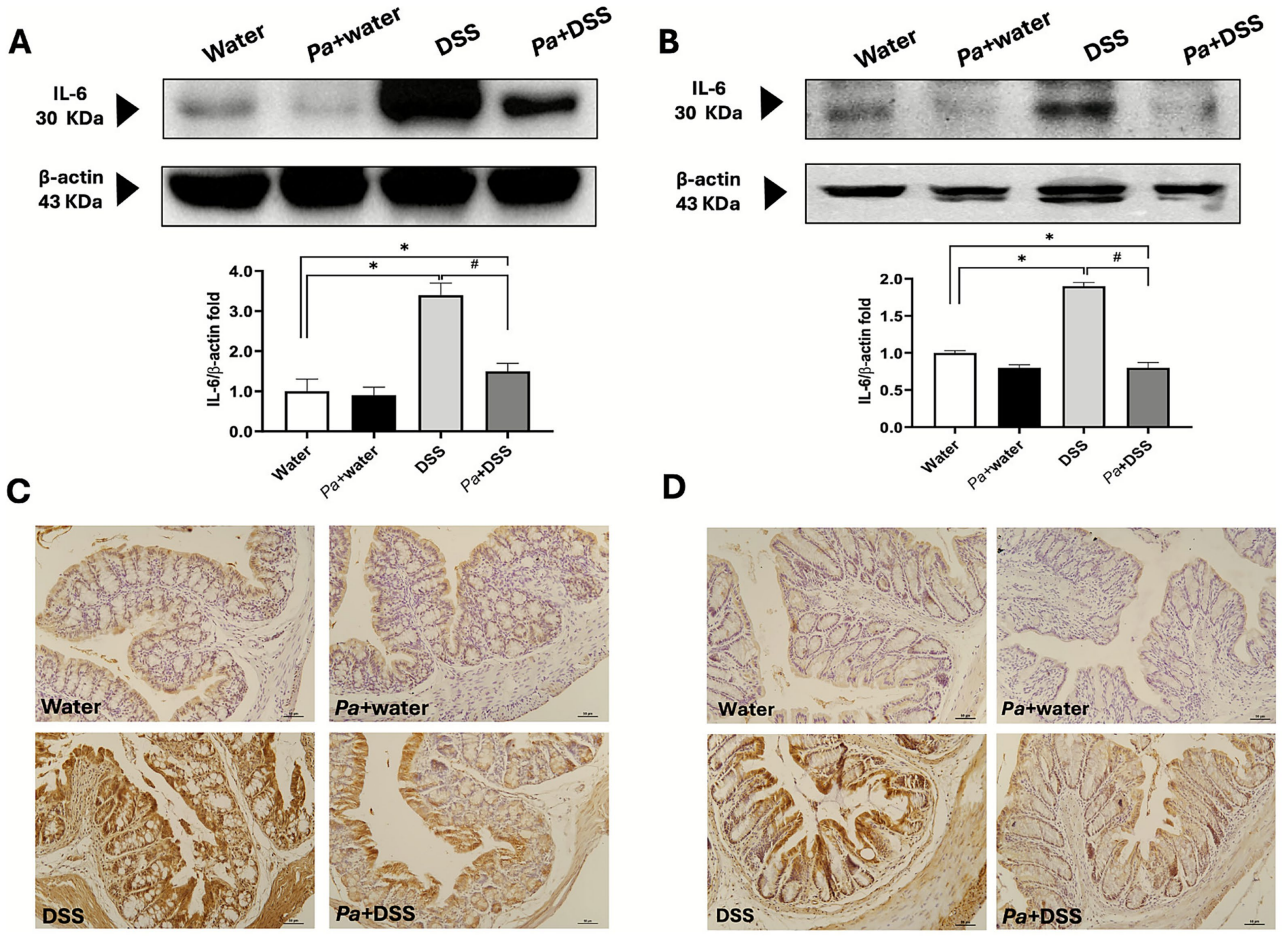
Interleukin 6 (IL-6) expression level evaluation in western blot and immunohistochemical technique. A specific antibody against IL-6 was immunoblotted on a lysate of proximal **(A)** and distal colon **(B)**. The relatives’ graphs indicate the ratio of the densitometric analysis of IL-6 bands and β-actin levels were used as reference loading control [bottom line of panels **(A,B)**]. Representative sections marked with anti-IL-6 of proximal **(C)** and distal colon **(D)** of mice belonging to the different experimental groups, as indicated at the bottom left of each figure. Calibration bar: 50 μm. Water: control mice; *Pa* + water: *P. acidilactici* 46A-pretreated control mice; DSS: 2.5% dextran-sulphate sodium treated mice; *Pa* + DSS: *P. acidilactici* 46A-pretreated DSS mice. The score values are the mean ± SEM. *p < 0.05 *vs* water control mice; ^#^*p* < 0.05 *vs.* DSS.

The morphological and functional alterations were highlighted by different morphological approaches, Masson’s trichrome staining (Figures 4A,B), Alcian Blue pH 2.5 and PAS staining to highlight the presence inside the goblet cells of mucopolysaccharides and glycoproteins, respectively (Supplementary Figure S6). More specifically, by a combination of these three staining procedures, it was possible to appreciate a loss in the cryptal architecture, the presence of the inflammatory infiltrate, thickening of *muscolaris mucosae* and depletion of goblet cells (Figures 4A,B). Both in the proximal (Supplementary Figures S6A–C) and distal (Supplementary Figures S6B–D) portions, a clear reduction of the Alcian Blue (Supplementary Figures S6A,B) and PAS (Supplementary Figures S6C,D) positive glands was observed in DSS groups, highlighting the severe damage of secretory cells involved in acidic mucopolysaccharides and glycoproteins mucus components. Moreover, it was possible to appreciate a morphological amelioration of the epithelium in the *Pa*+ DSS groups (Figures 4A,B), suggested by a maintenance in the cryptal structure and by a non-significant depletion in the number of goblet cells (Supplementary Figure S6); the inflammatory infiltrate, prominent in the DSS groups, was approximately absent in the *Pa*+ DSS samples. The data related to the histological score for each experimental group were reported in Figures 4C,D and suggest that the distal colon has been damaged to a greater extent by DSS compared to the proximal portion. In both cases, supplementation with *P. acidilactici* 46A resulted in a significant reduction in histological damage. In absolute terms, the damage recorded was evident in all colonic tract. However, the distal portion was more damaged, with an evident thickening of the muscularis mucosae (Figures 4A,B). Despite the distal *Pa*+ DSS scoring being higher than the proximal *Pa*+ DSS scoring, the percentage of improvement in the proximal and distal colon was quite similar, considering the initial differences in the damage between the two DSS groups (Figures 4C,D). In addition to morphological staining, immunohistochemical analysis was performed to evaluate the pro-inflammatory microenvironment at the level of the colonic mucosa in the different experimental groups. More specifically, the study was conducted against IL-1*β* and IL-6, the pro-inflammatory cytokines that play a key role in the regulation of immune and inflammatory responses (Le Berre et al., 2023). As expected, the samples from mice treated with DSS induced an increase in expression of pro-inflammatory cytokines. Considering the protein expression characterization by WB of IL1-β a clear immunoreaction lanes were detected at 30 and 17 KDa that highlight, respectively, the precursor and mature isoform of that cytokine. The WB results revealed a significant increment, respect to the control group, of IL-1β expression in the groups of mice that receive DSS and *Pa*+ DSS respect to the control one, both in the proximal and distal colon lysates (Figures 5A,B). Moreover, the administration of *P. acidilactici* 46A seems to reduce in a statistically significant way the expression of this interleukin in the proximal colon lysate (Figure 5A). Sections of the proximal (Figure 5C) and distal colon (Figure 5D) processed for the IL-1β immunohistochemistry revealed an increase of expression in the mice of DSS groups, both in the mucosa and in the submucosal layer. The reaction was lower in the *Pa*+ DSS group (Figures 5C,D). Similar results were also obtained using western blot technique, and observed for the immunodetection of IL-6 (Figure 6). The WB revealed a band around 30 KDa with a clear increment of in the DSS groups both in the proximal and distal colon (Figures 6A,B). Moreover, a reduction of IL-6 expression was evident in the colon lysate of mice of *Pa*+ DSS group respect to the control one (Figures 6A,B). The WB results were confirmed by immunohistochemistry in which a strong IL-6 immunoreaction was detected in the all-colonic tract of the animals treated with DSS, and an attenuated immunostaining was registered in the DSS group with preventive probiotic supplementation (Figures 6C,D).

### 3.4 Mice gut microbiota changes during treatments and short chain fatty acid analysis

Gut microbiota changes were analyzed through diversity and taxonomic profiling of sequencing data obtained from the V3–V4 regions of the 16S rRNA gene. Comparative analyses were divided into two parts.

**Figure 7:**
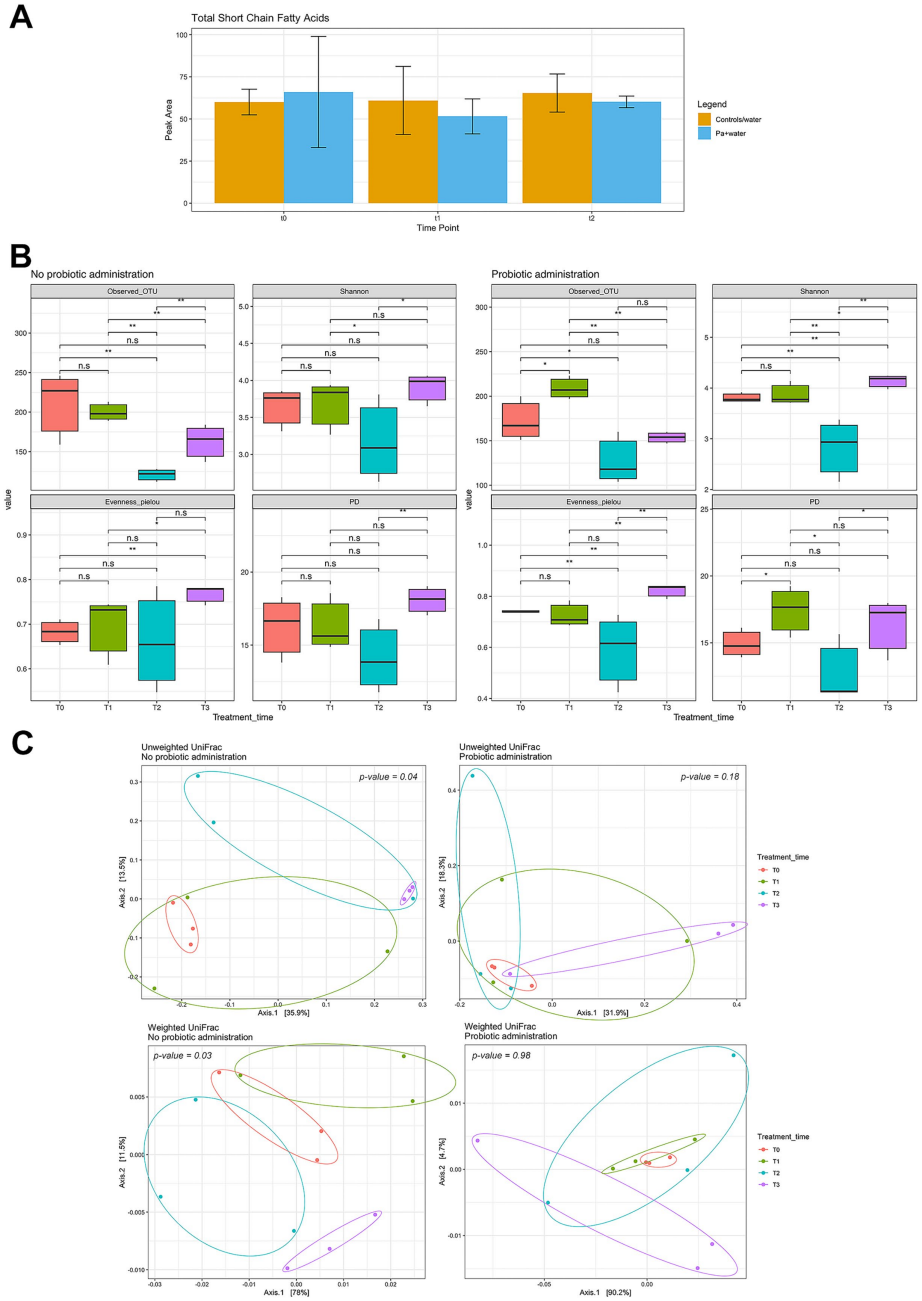
**(A)** Total SCFA concentration in fecal samples from mice in the control/water and *Pa* + water groups. **(B)** Effects of *P. acidilactici* 46A administration on gut microbiota composition during DSS-induced colitis, assessed by alpha diversity analysis. ^*^p < 0.05, ^**^p < 0.01 between the four time points. **(C)** Beta diversity analysis of the same samples shown in **(B)**. *p*-values derived from the PERMANOVA statistical test are reported directly on the panels. The “Probiotic administration” panel refers to samples from mice in the *Pa* + DSS group, whereas “No probiotic administration” refers to samples from mice in the DSS group.

The first comparison included control mice (“water”) and mice treated with probiotics alone (“*Pa* + water”) at three time points defined in the experimental design (Figure 1; T0, T1, and T2), as T3 was identical to T2. Alpha diversity analysis did not reveal significant differences among treatments (Supplementary Figure S7). Similarly, beta diversity analysis did not show clear clustering, except for T0 samples collected before probiotic supplementation (*p* < 0.05), and was therefore not reported. A corresponding analysis of these samples was performed to determine short-chain fatty acid (SCFA) composition. A slight decrease in total SCFA concentration was observed after one week of probiotic administration (T1), possibly indicating a temporary imbalance in microbiota composition, followed by a slight increase during the second period of treatment (T2). In contrast, the control group maintained a relatively stable SCFA content, with only a slight increase during the last period. No statistically significant differences between groups were detected (Figure 7A).

The second comparison included mice treated with DSS and *Pa* + DSS (lines 3 and 4 of Figure 1) and was performed at four time points (T0–T3). In this analysis, alpha and beta diversity, changes at phylum and genus levels, and co-occurrence patterns at both levels were evaluated. Alpha diversity analysis revealed statistically significant changes during DSS treatment (DSS group, T2). The number of observed OTUs indicated higher species richness in the *Pa* + DSS group both after probiotic administration and following DSS treatment compared with the DSS group. In addition, the Shannon and Pielou evenness indices suggested a more even distribution of bacterial species in the *Pa* + DSS group (Figure 7B).

Beta diversity analysis (Figure 7C) compared fecal microbiota from DSS-treated mice (line 3 in Figure 1) and Pa + DSS–treated mice (line 4 in Figure 1). Significant separation of clusters (*p* < 0.05) was observed in the left panels for DSS-treated mice without probiotic administration, indicating DSS-induced disruption of gut microbiota composition. In contrast, no significant clustering was observed in mice pretreated with probiotics (*p* > 0.1), supporting a preventive protective effect of *P. acidilactici*.

**Figure 8:**
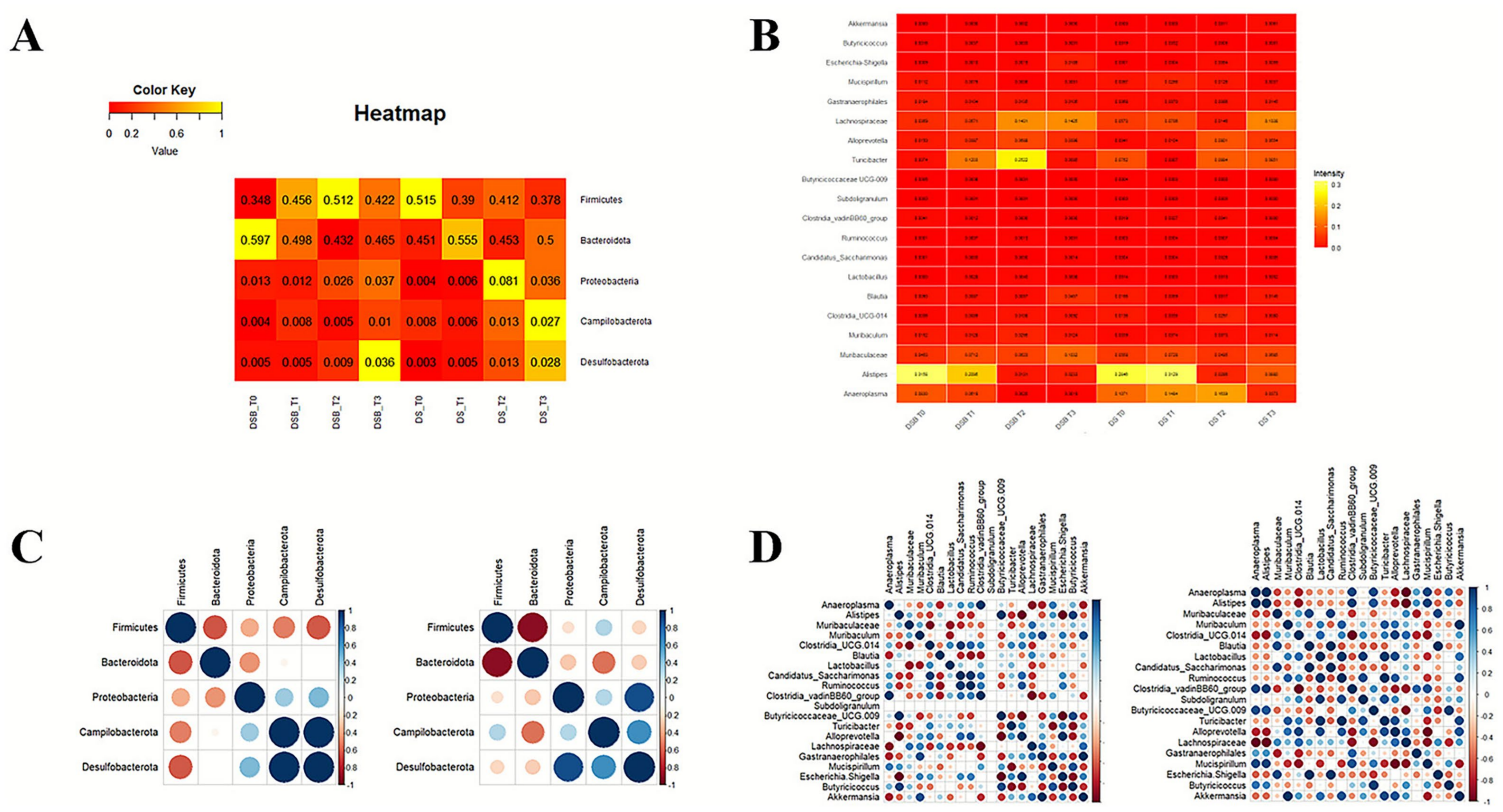
**(A)** Heatmap representing changes in phylum composition of microbiota of *Pa*+DSS and DSS group through four time points. **(B)** Heatmap representing bacteria groups composition of microbiota in *Pa*+DSS and DSS groups through four timepoints. **(C)** Phylum co-occurrence dot-plot DSS group (on the left) and *Pa*+DSS (on the right). **(D)** Bacteria genera and families co-occurrence dot-plot for DSS (on the left) and *Pa*+DSS (on the right) mice groups.

To further characterize gut microbiota composition, the five most abundant phyla were analyzed: *Firmicutes*, *Bacteroidota*, *Proteobacteria*, *Campylobacterota*, and *Desulfobacterota*. The relative abundance of *Firmicutes* and *Desulfobacterota* was notably increased in the *Pa* + DSS group. Moreover, the *Pa* + DSS group showed a faster restoration of the *Firmicutes/Bacteroidota* balance at T3, after cessation of DSS treatment and resumption of water administration (Figure 8A), compared with the DSS group. In addition, the 20 most abundant bacterial groups are shown in the heat map in Figure 8B. Notably, the *Pa* + DSS group exhibited a lower relative abundance of bacterial taxa commonly associated with inflammation and colitis, and a higher abundance of taxa associated with gut health and/or SCFA production, compared with the DSS group.

To better understand the influence and dynamics of bacterial interactions during DSS-induced colitis, co-occurrence analyses were performed at both phylum and genus levels (Figures 8C,D). Co-occurrence patterns among all analyzed phyla were significantly altered by DSS treatment, suggesting a strong impact on inter-phyla interactions. In contrast, the *Pa* + DSS group (Figure 8C) showed a general trend toward the maintenance of stronger co-occurrence relationships among phyla. Co-occurrence analyses of 20 bacterial genera and families (Figure 8D) further highlighted distinct interaction patterns between the DSS and *Pa* + DSS groups.

## 4 Discussion

In this study, we analyzed the whole genome of *P. acidilactici* 46A to evaluate its functional potential. The genome spans 2,086,432 bp and includes 2,073 genes, slightly larger than the *P. acidilactici* PMC65 reference genome (2,044,083 bp, 2,008 genes). Five CRISPR sequences were identified, consistent with their role in antiphage defence and prophage inhibition in lactobacilli (Pei et al., 2021). COG annotations revealed a strong representation of genes involved in carbohydrate metabolism, membrane transport, nucleotide metabolism, and translation. The prominence of carbohydrate metabolism genes is particularly notable and may contribute to gut health benefits. Moreover, a strong antimicrobial property of *P. acidilactici* against different strains of *Listeria monocytogenes*, vancomycin-resistant *Enterococcus* strains, was documented. Thanks to the presence of pediocin PA-1 and the production of benzoic acid, 2-hydroxyisocaproic acid, *β*-phenyl-lactic acid, *α*-hydroxybutyric acid and 1,3-butanediol, two strains *P. acidilactici* ST3522BG and *P. pentosaceus* ST3633BG, showed the ability to inhibit the growth of fungi, such as *Alternaria alternata*, *Aspergillus flavus*, *Aspergillus niger*, *Cladosporium sphaerospermum*, *Penicillium chrysogenum* and *Penicillium expansum* (Fugaban et al., 2022).

Antibiotic resistance profiling (Supplementary Table S1) showed *P. acidilactici* 46A was resistant to several antibiotics. Resistance to β-lactams (penicillin G, ampicillin), chloramphenicol, and erythromycin is generally uncommon in *P. acidilactici*, although strain variability has been reported (Danielsen et al., 2007). In agreement with Singla et al. (2018), *P. acidilactici* 46A displayed intrinsic resistances to vancomycin, aminoglycosides, nalidixic acid and sulfametoxazole-trimethoprim, traits considered part of the natural phenotype of *Pediococcus* species and generally regarded as non-transferable and not posing a safety risk. (Singla et al., 2018). In *P. acidilactici* 46A resistance genes were located chromosomally, and no plasmids were detected. These findings are consistent with previous studies highlighting the genetic diversity and adaptability of *P. acidilactici* strains, particularly with respect to probiotic-related traits (Aziz et al., 2025). Chromosomal localization of resistance genes, especially in the absence of plasmids or identifiable mobile genetic elementss, substantially reduces the likelihood of horizontal gene transfer and is not considered a safety concern under Qualified Presumption of Safety (QPS)/EFSA criteria. Acquired resistance determinants such as *tet(M)*, *erm(B)*, and *lnu(A)* have been found in a minority of *Pediococcus* strains and require careful assessment in safety evaluations (Chaichana et al., 2025; Hazards et al., 2023), Consistent with literature-based risk assessment criteria distinguishing intrinsic from acquired resistance, *P. acidilactici* 46A showed phenotypic resistance to tetracycline without harboring known tetracycline resistance genes such as *tet(M)*. This phenotype is likely attributable to intrinsic physiological mechanisms, including reduced membrane permeability, non-specific efflux activity, ribosomal features, or growth-related tolerance. As these mechanisms are chromosomally encoded and non-mobile, they do not represent a risk for horizontal gene transfer and should not be interpreted as acquired antimicrobial resistance in the absence of mobile resistance genes or mobile genetic elements according to the European Food Safe Authority concerning the antimicrobial resistance gene criteria.

In the DSS-induced colitis mouse model, clinical assessments (body weight, stool consistency, colitis score) confirmed successful colitis induction. Although body weight loss was modest (<10%), the DSS treated mice showed significantly higher colitis scores than controls. Mice pretreated with *P. acidilactici* 46A (*Pa*+ DSS) displayed reduced colitis severity. Colon length, which typically shortens under colitis conditions, was significantly reduced in DSS-treated mice but preserved in the *Pa*+ DSS group, in line with previous studies (Zou et al., 2023).

Histological analyses revealed severe mucosal damage in DSS-treated mice, including crypt loss, inflammatory infiltrate, and goblet cell depletion. In contrast, *Pa*+ DSS mice showed improved epithelial architecture and preserved goblet cell morphology. Mucus staining (Alcian Blue pH 2.5 and PAS) further supported enhanced mucosal integrity and reduced goblet cell loss in *Pa*+DSS mice. These findings are consistent with previous studies demonstrating that probiotics can restore mucosal barriers (Bian et al., 2019; Chae et al., 2018; Ding et al., 2019).

The pathogenesis of colitis involves disruption of epithelial tight junctions and immune activation, leading to cytokine release such as IL-6, IL-1β, TNF-*α*, and COX-2 (Le Berre et al., 2023). Western blot and immunohistochemical analyses confirmed elevated IL-1β and IL-6 expression in DSS-treated colons. In *Pa*+ DSS mice, the expression of these interleukins was reduced, indicating an attenuated inflammatory response. This observation is in line with reports showing that probiotics downregulate pro-inflammatory cytokines and promote mucosal healing (Kim et al., 2012; Celiberto et al., 2015; Kim et al., 2023).

Microbiota analysis showed a transient increase in OTUs at T1, possibly reflecting initial dysbiosis. Beta diversity analysis revealed distinct microbial clustering between DSS and *Pa*+ DSS groups, consistent with probiotic-induced microbiota modulation (Kim et al., 2012; Mendes et al., 2018). Taxonomic changes included increased *Blautia* spp. (a butyrate producer), reduced abundance of *Eubacterium* spp., and suppression of *Eu. coprostanoligenes*. Clostridiales vadin BB60 group, associated with immune dysregulation, increased in DSS-treated mice but were less abundant in *Pa*+ DSS mice.

The abundance of *Lachnospiraceae* increased with *P. acidilactici* 46A treatment with respect to only water treatment. This family is known for SCFA production and is generally considered beneficial, although some members are associated with metabolic disorders (Zhang et al., 2019). No significant differences in SCFA levels were observed between *Pa*+water and control groups, possibly reflecting microbial adaptation or stress-related effects following probiotic administration (Maltz et al., 2019).

At the phylum level, *Pa*+ DSS mice showed higher Firmicutes and lower Bacteroidota compared to DSS mice, with a more balanced Firmicutes/Bacteroidota (F/B) ratio post-treatment, a parameter often associated with gut health (Stojanov et al., 2020). Proteobacteria, commonly associated with inflammation, were elevated in DSS mice but reduced in *Pa*-treated groups (Wang et al., 2020). Similarly, Campylobacterota increased in DSS-treated mice but were lower in *Pa*-treated groups and absent in *Pa*+water mice, further supporting a probiotic-mediated protective effect.

Desulfobacterota abundance increased in both DSS and *Pa*+DSS mice, consistent with microbiota imbalance and immune activation (Xiao et al., 2021). The opportunistic *Anaeroplasma* genus enriched in DSS mice and decreased in *Pa*+ DSS mice. Though previously considered pathogenic, emerging data suggest that *Anaeroplasma* may have anti-inflammatory effects under specific conditions (Beller et al., 2019, 2020). M*uribaculaceae* abundance increased in *Pa*-treated groups. This family has been associated with colitis recovery (Huang et al., 2022) and mucosal protection (Wang et al., 2022). Beneficial shifts were also observed in *Clostridia UCG-014*, *Lactobacillus*, *Candidatus Saccharimonas*, *Ruminococcus* spp., and butyrate-producing Butyricicoccaceae UCG-009. *Subdoligranulum* spp., absent in DSS mice, was present in *Pa*-treated mice, supporting its proposed probiotic role and contribution to metabolic health (Luo et al., 2024). *Turicibacter* spp., which have been reported to reduce inflammation and metabolic stress via insulin signaling (Guo et al., 2022) were more abundant in *Pa*+DSS mice. Lachnospiraceae abundance increased only in *Pa*+DSS, further indicating the beneficial effects of probiotic administration (Peng et al., 2020). Finally, species co-occurrence analysis revealed preserved microbial network stability in *Pa*+DSS mice, in contrast to the disrupted community in DSS-only mice. This finding further supports the protective and restorative role of *P. acidilactici* 46A in maintaining gut homeostasis under inflammatory stress.

Despite the promising results, some limitations of the present study should be acknowledged. First, the probiotic administration was limited to a relatively short pre-treatment period; longer-term supplementation may be required to fully evaluate the stability and persistence of the observed protective effects on gut inflammation and microbiota composition. Second, probiotics were administered prior to DSS exposure but not during DSS treatment. Although this approach was chosen to avoid potential confounding effects related to DSS-induced diarrhea and reduced oral intake, future studies could explore alternative dosing strategies to assess whether continued administration during inflammatory challenge further enhances therapeutic efficacy. In addition, only a single probiotic dose was tested in this study; evaluation of different concentrations could help define dose–response relationships and optimize treatment conditions.

## 5 Conclusion

This study provides integrated *in vitro*, genomic, and *in vivo* evidence supporting the probiotic potential of *P. acidilactici* 46A. Functional genome analysis revealed a repertoire of genes associated with carbohydrate metabolism, stress response, and antimicrobial activity, supporting its adaptation to the gastrointestinal environment. In the DSS-induced colitis mouse model, oral administration of *P. acidilactici* 46A attenuated disease severity, as demonstrated by reduced colitis scores, preservation of colon length, improved histological architecture, and decreased expression of the pro-inflammatory cytokines like IL-1β and IL-6.

Microbiota analyses further showed that *P. acidilactici* 46A modulated gut microbial composition during inflammatory stress, promoting higher microbial richness, preservation of beta diversity structure, and restoration of beneficial bacterial taxa, including SCFA-producing and mucosa-associated groups. The probiotic treatment also preserved microbial network stability, counteracting the dysbiosis and interaction disruption induced by DSS. These findings suggest that the protective effects of *P. acidilactici* 46A are mediated through both immune modulation and maintenance of gut microbiota homeostasis.

Importantly, whole-genome sequencing indicated that all detected antibiotic resistance genes were chromosomally encoded and that no plasmids were present, supporting the biosafety profile of this strain. While additional targeted safety assessments are warranted, these genomic features strengthen the candidacy of *P. acidilactici* 46A for further translational development.

Overall, *P. acidilactici* 46A emerges as a promising probiotic candidate for preventing or mitigating intestinal inflammation. Future studies should focus on identifying the specific metabolic pathways, microbial interactions, and bioactive compounds underlying its anti-inflammatory and microbiota-modulating effects, as well as validating its efficacy in additional preclinical models and human studies.

## Data Availability

The datasets presented in this study can be found in online repositories. The names of the repository/repositories and accession number(s) can be found at: https://www.ncbi.nlm.nih.gov/, PRJNA1344905.

## Footnotes

1
https://github.com/ctSkennerton/minced

2
https://github.com/moshi4/COGclassifier
