# Supplementary material for: Evaluation of probiotic properties and genome analysis of the new Pediococcus acidilactici strain 46A isolated from Chinese young adults: in vitro and in vivo studies
Source: Front Microbiol. 2026 Feb 16;17:1725578. doi: 10.3389/fmicb.2026.1725578 (PMC12951473; doi:10.3389/fmicb.2026.1725578)
Supplement: Supplementary file 1 [file Data_sheet_1.docx]

Supplementary Material

**Table 1S.** Phenotypic antimicrobial susceptibility of *Pediococcus acidilactici* 46A, where S stands for susceptible and R for resistant.

| **Antibiotics** | **Antibiotic Concentrations** | **Antibiotic Pattern** |
| --- | --- | --- |
| **Penicillins** | | |
| Benzylpenicillin G | P-1UI | S |
| Ampicillin | AMP-10µg | S |
| Piperacillin | PRL-30µg | S |
| Oxacillin | OX-1µg | R |
| **Penicillins+beta-lactamase inhibitors** | | |
| Amoxicillin and clavulanic acid | AUG-30µg | S |
| **Carbapenems** | | |
| Imipenem | IMI-30µg | R |
| **I gen. cephalosporins** | | |
| Cefadroxil | CDX-30µg | R |
| Cephalexin | CL-30µg | S |
| **II gen. cephalosporins** | | |
| Cefoxitina | FOX-30µg | R |
| Cefixime | CFM-30µg | R |
| Ceftiofur | FUR-30µg | S |
| **III gen. cephalosporins** | | |
| Ceftazidime | CAZ-30µg | R |
| **IV gen. cephalosporins** | | |
| Cefquinome | CEQ-30µg | S |
| **Monobactams** | | |
| Aztreonam | ATM-30µg | R |
| **Fluoroquinolones** | | |
| Ciprofloxacin | CIP-5µg | R |
| Enrofloxacin | ENR-30µg | R |
| Norfloxacin | NOR-10µg | R |
| Nalidix acid | NA-30µg | R |
| **Aminoglycosides** | | |
| Amykacin | AK-30µg | R |
| Gentamicin | CN-10µg | R |
| Tobramycin | TOB-10µg | R |
| **Glycopeptides** | | |
| Vancomycin | VA 0.5-32 µg/mL | R |
| Teicoplanin | TEC 0.5-32 µg/mL | R |
| **Macrolides** | | |
| Azithromycin | AZM-15µg | R |
| Erythromycin | E-15µg | S |
| Streptomycin | S-300µg | R |
| Spiramycin | SP-100µg | S |
| **Lincosamides** | | |
| Clindamycin | CD-2µg | S |
| **Streptogramins** | | |
| Quinupristin-dalfopristin | QD-15µg | R |
| **Tetracyclines** | | |
| Tetracycline | TE-30µg | R |
| Tigecycline | TGC-15µg | S |
| **Polymyxins** | | |
| Colistin | CS 0.016-256µg/mL | R |
| Polymyxin B | PB-300IU | R |
| **Oxazolidones** | | |
| Linezolid | LNZ-10µg | S |
| **Folate pathway inhibitors** | | |
| Sulfametoxazole and Trimethoprim | SXT-25µg | R |
| **Amphenicols** | | |
| Florfenicol | FFC-30µg | S |
| **Phosphonic antibiotics** | | |
| Fosfomycin | FOS-200µg | R |
| **Rifamycins** | | |
| Rifampicin | RD-5µg | R |
| **Lipopeptydes** | | |
| Daptomycin | DAP-30µg | R |
| **Mupirocins** | | |
| Mupirocin | MUP-200µg | R |
| **Nitrofurans** | | |
| Nitrofurantoin | F-100µg | S |

**Table 2S:** Identification of the nine selected strains.

| **STRAIN’S CODE** | **ID. METHOD** | **IDENTIFICATION** |
| --- | --- | --- |
| **24H** | MALDI-TOF MS tec.* | *Lactiplantibacillus plantarum* |
| **38B** | MALDI-TOF MS tec.* | *L. plantarum* |
| **48A** | MALDI-TOF MS tec.* | *L. plantarum* |
| **22A.1** | MALDI-TOF MS tec.* | *Lacticaseibacillus paracasei ssp. paracasei* |
| **22A.2** | MALDI-TOF MS tec.* | *Limosilactobacillus fermentum* |
| **22A.3** | MALDI-TOF MS tec.* | *Lacticaseibacillus rhamnosus* |
| **26A** | MALDI-TOF MS tec.* | *L. paracasei ssp. paracasei* |
| **44A** | MALDI-TOF MS tec.* | *Pediococcus acidilactici* |
| **46A** | MALDI-TOF MS tec.* | *P. acidilactici* |


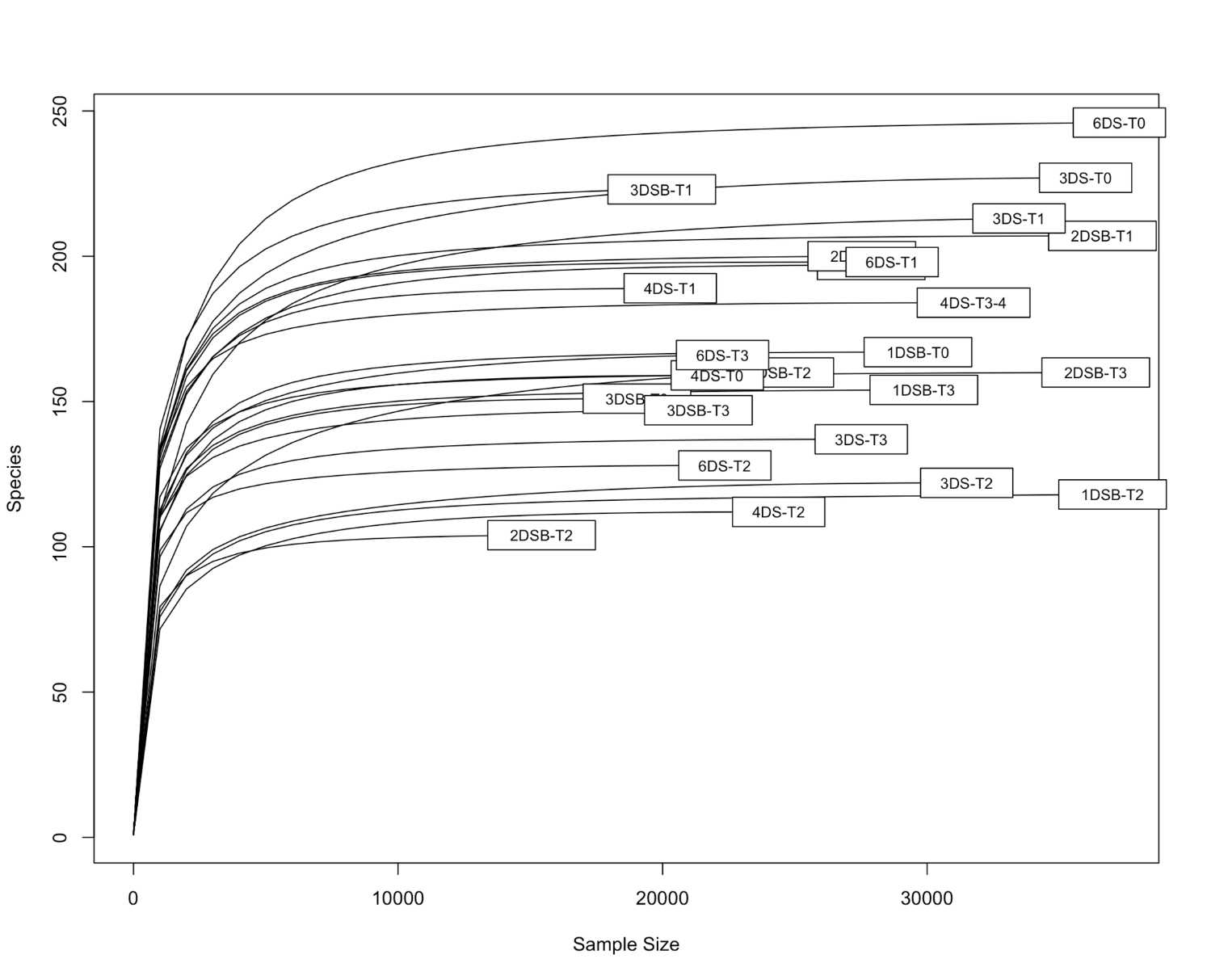


Figure 1S: Refraction curves


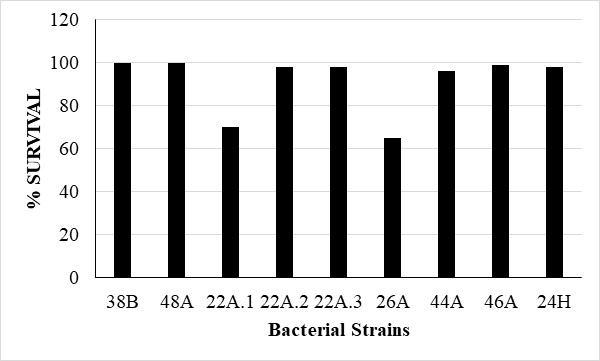


**Figure 2S**. Survival (as %) of the nine tested strains after 5 hours at acid condition.

**A
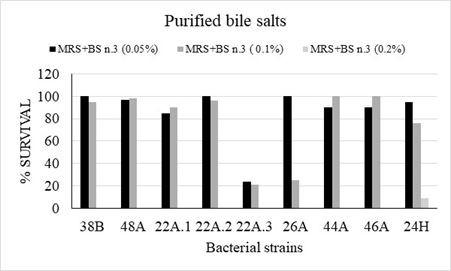
** **B**
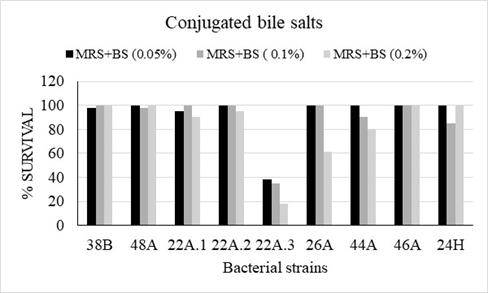


**Figure 3S:** Percentage of strain survival in bile salts-enriched media. Purified bile salts (A) and conjugated bile salts (B).


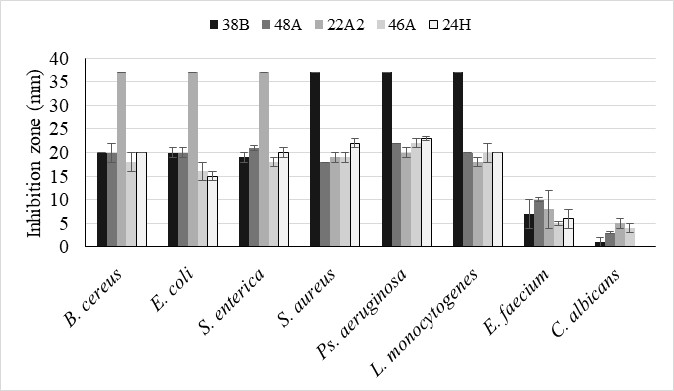


**Figure 4S.** Antimicrobial activity assay. The inhibition zone is expressed in mm (values are mean of duplicates; the *s.d*. is not always visible). *B. cereus* DSM 345, E. *coli* ATCC 13706, *S.* *enterica* DSM 14221, *S. aureus* ATCC 25923, *P.* *aeruginosa* DSM 1117, *L. monocytogenes* 306, *E. faecium* DSM 13590, *C. albicans* ATCC 10261.


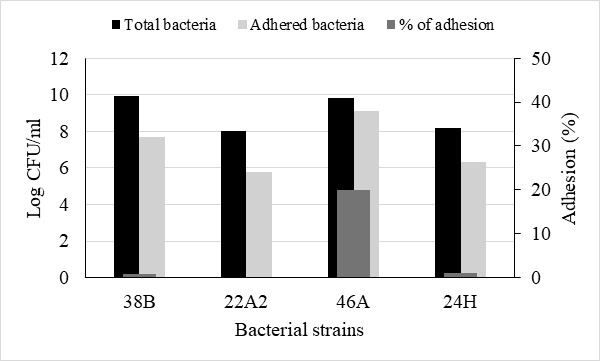


**Figure 5S.** Adhesion test of candidate probiotic strains on intestinal epithelial HT-29 cell lines. Total bacteria, adhered bacteria and % of adhesion are reported. Values are means of duplicates; the low values of *s.d*. are not visible.


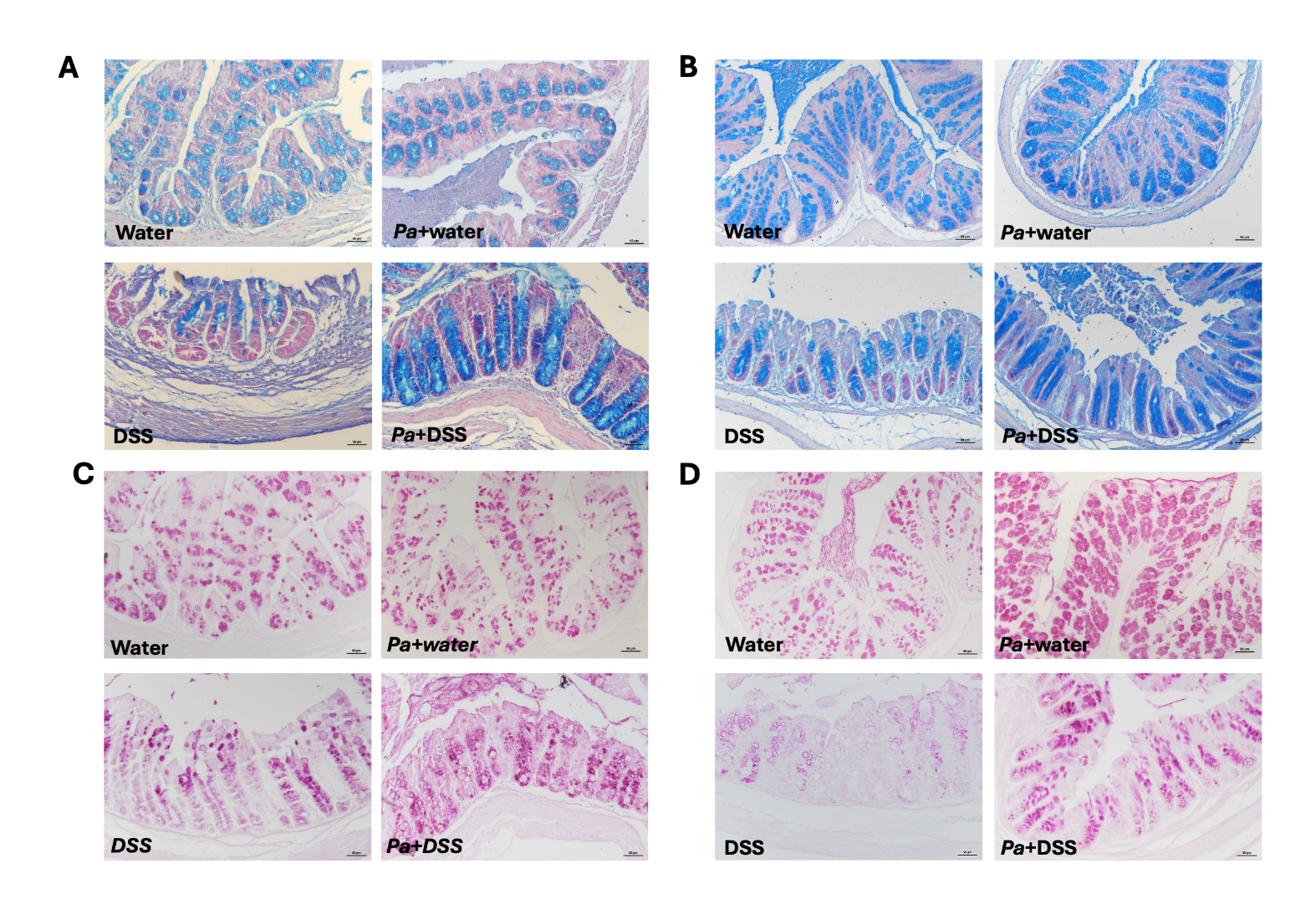


**Figure 6S:** Representative sections stained with Alcian blue pH 2.5 of proximal colon **(A)** and distal colon **(B)** and representative sections stained with periodic acid Schiff staining (PAS) of proximal colon **(C)** and distal colon **(D)** of mice belonging to the different experimental groups. Calibration bar: 50 µm. Water: control mice; *Pa*+ water: *P. acidilactici* 46A – pretreated control mice; DSS: 2.5% dextran-sulphate sodium treated mice; *Pa*+ DSS: *P. acidilactici* 46A – pretreated DSS mice.

**
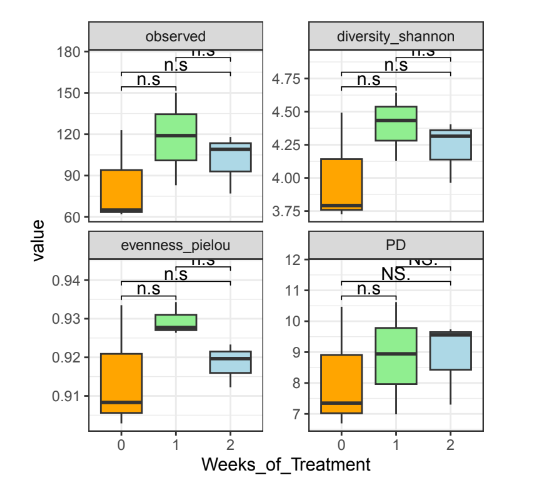
**

**Figure 7S:** Alpha diversity comparison between “Water” and “*Pa*+Water” groups through three time points.
